# Cloudberry (*Rubus chamaemorus* L.) Supplementation Attenuates the Development of Metabolic Inflammation in a High-Fat Diet Mouse Model of Obesity

**DOI:** 10.3390/nu14183846

**Published:** 2022-09-17

**Authors:** Toini Pemmari, Mari Hämäläinen, Riitta Ryyti, Rainer Peltola, Eeva Moilanen

**Affiliations:** 1 The Immunopharmacology Research Group, Faculty of Medicine and Health Technology, Tampere University and Tampere University Hospital, 33014 Tampere, Finland; 2 Bioeconomy and Environment, Natural Resources Institute Finland, 96100 Rovaniemi, Finland

**Keywords:** cloudberry, *Rubus chamaemorus*, high-fat diet, ellagitannins, metabolic inflammation, hypercholesterolemia, hyperglycemia

## Abstract

Metabolic diseases linked to obesity are an increasing problem globally. They are associated with systemic inflammation, which can be triggered by nutrients such as saturated fatty acids. Cloudberry is rich in ellagitannin and its derivatives, which are known to have anti-inflammatory properties. In the present study, a high-fat-diet-induced mouse model of obesity was used to study the effects of air-dried cloudberry powder on weight gain, systemic inflammation, lipid and glucose metabolism, and changes in gene expression in hepatic and adipose tissues. Cloudberry supplementation had no effect on weight gain, but it prevented the rise in the systemic inflammation marker serum amyloid A (SAA) and the hepatic inflammation/injury marker alanine aminotransferase (ALT), as well as the increase in the expression of many inflammation-related genes in the liver and adipose tissue, such as *Mcp1*, *Cxcl14*, *Tnfa*, and *S100a8*. In addition, cloudberry supplementation impeded the development of hypercholesterolemia and hyperglycemia. The results indicate that cloudberry supplementation helps to protect against the development of metabolic inflammation and provides partial protection against disturbed lipid and glucose metabolism. These results encourage further studies on the effects of cloudberry and cloudberry-derived ellagitannins and support the use of cloudberries as a part of a healthy diet to prevent obesity-associated metabolic morbidity.

## 1. Introduction

Metabolic diseases such as atherosclerotic disease, type II diabetes, and non-alcoholic fatty liver disease (NAFLD) are associated with obesity and metabolic inflammation. Excess intake of saturated fatty acids, cholesterol, and sugars contributes to the development of these adverse conditions [1]. The most important immune cells in metabolic inflammation are monocytes and macrophages [1]. Dietary saturated fatty acids activate macrophages in adipose and hepatic tissues [2,3]. Their activation leads to the production of proinflammatory cytokines, induction and maintenance of metabolic inflammation, and development of insulin resistance [4]. In addition, excess intake of saturated fatty acids causes hypercholesterolemia, which is also a major risk factor for atherosclerotic diseases [5].

Prevention of metabolic inflammation with nutrition is a tempting approach to prevent the development of the comorbidities of obesity and a Western-style high-fat diet. Vegetables, fruit, and berries rich in polyphenolic compounds have shown promising effects on obesity-related adverse metabolic effects and metabolic inflammation in experimental models and human studies [6]. In the present study, we were interested in the potential effects of wild cloudberry, which remains mostly unexplored as compared to many other wild berries.

Cloudberry (*Rubus chamaemorus*) is a wild boreal plant that is part of the traditional diet in Nordic countries, Russia, and Canada. Yellow cloudberry berries have a refined taste and unique polyphenol composition compared to wild berries such as bilberry (*Vaccinium myrtillus*) and lingonberry (*Vaccinium vitis-idaea*) [7,8,9]. The main polyphenols in cloudberries are ellagitannins, whereas bilberry and lingonberry are rich in anthocyanins and proanthocyanidins, respectively [8,10].

In in vitro studies, ellagitannins have been reported to have anti-inflammatory and metabolic effects, which can be expected to inhibit metabolic inflammation and adverse metabolic effects induced by obesity and a high-fat diet. Ellagic acid was found to impede the formation of foam cells [11], which is a significant pathophysiological mechanism in atherosclerotic disease [5]. Ellagic acid was also reported to inhibit the expression of Toll-like receptor 4 [12], an important mediator of the proinflammatory actions of fatty acids and many other factors [2,3]. Accordingly, the activation of the inflammatory transcription factor NF-κB was found to be inhibited by ellagitannins [13], as well as that of the production of cytokines IL-6 and TNF-α and many other proinflammatory mediators [14,15]. These findings led us to hypothesize that cloudberry may have bioactivities that could inhibit high-fat-diet-induced metabolic inflammation and its consequences in obesity.

In the present study, we examined the effects of air-dried Arctic cloudberry on high-fat-diet-induced obesity in a mouse model. We discovered that cloudberry supplementation prevented the increase in inflammatory markers and impeded the development of hypercholesterolemia and hyperglycemia without a significant effect on weight gain.

## 2. Materials and Methods

### 2.1. Mice and Study Design

Male C57BL/6N mice (*n* = 72) at the age of eight weeks were divided into six groups of twelve mice. Two mice were housed in one cage throughout the study. The mice were maintained on a low-fat diet (LF, 10% of energy from fat), high-fat diet (HF, 46% of energy from fat), or cloudberry-supplemented high-fat diet (HF+CLB, 20% of weight from air-dried cloudberry powder). After six and twelve weeks, a group of twelve mice in each treatment arm was terminated, and samples were collected for analysis as described below.

The cloudberry powder was produced from Finnish cloudberries, where approx. 700 g of fresh berries produce an average of 100 g of powder (Kiantama Oy, Suomussalmi, Finland). The diets were custom-made in collaboration with the manufacturer (Research Diets Inc., New Brunswick, NJ, USA) to match their fiber, protein, and other ingredients for the composition of the cloudberry powder (Table 1).

The weights of the mice and the food intake were recorded weekly. One week before the end of the study (at weeks five and eleven), an intraperitoneal glucose tolerance test (IPGTT) was conducted. At the end of the study (at weeks six and twelve), serum and tissue samples were collected for analyses. The mice were housed in the preclinical facility of Tampere University under stable conditions (light/dark cycle 12 h, temperature 22 ± 1 °C, and humidity 50–60%). Food and water were provided ad libitum. The study was approved by the National Animal Experimental Board of Finland (ESAVI/984/04.10.07/2018). The experiments were conducted in accordance with EU legislation (Directive 2010/63/EU).

### 2.2. Intraperitoneal Glucose Tolerance Test

The IPGTT was performed at weeks five and eleven, i.e., one week before the end of the study. After a morning fast of six hours, the fasting glucose was measured from the peripheral tail vein (Contour Next One, Oy Diabet Ab, Lemu, Finland). Sterile-filtered glucose in phosphate-buffered saline (2 g/kg, Sigma-Aldrich, St. Louis, MO, USA) was injected intraperitoneally. Blood glucose was measured 30, 60, 90, 120, and 180 min after the injection.

### 2.3. Blood Samples and Analyses

Before the end of the study, at weeks six and twelve, the mice were fasted for six hours in the morning. Under isoflurane anesthesia, blood was collected via cardiac punction, and euthanasia was performed by cervical dislocation. Blood samples were incubated for 30 min at room temperature and centrifuged for 15 min at 1500× *g*, and the obtained serum was immediately frozen and stored at −80 °C. Serum cholesterol and triglyceride levels as well as alanine aminotransferase (ALT) activity were measured by fluorometric assays (Abcam, Cambridge, UK). Serum insulin (Mercodia Ltd., Uppsala, Sweden), serum amyloid A (SAA), leptin, resistin, adipsin, and adiponectin (R&D Systems Europe Ltd., Abingdon, UK) were measured by enzyme-linked immunoassays (ELISAs). The detection limits were 33 pmol/L for insulin, 62.5 ng/L for SAA, 7.8 ng/L for leptin and resistin, 375 ng/L for adipsin, and 15.6 ng/L for adiponectin.

### 2.4. RNA Extraction and Reverse Transcription Polymerase Chain Reaction

RNA Later© (Ambion, Thermo Fisher Scientific, Waltham, MA, USA) was used to store the liver and epididymal fat samples immediately after collection. For RNA extraction, 25–30 mg of liver tissue or 125–135 mg of epididymal fat tissue was cut into small pieces. The liver tissue was homogenized with Qiashredder (Qiagen Inc., Hilden, Germany), and RNA was extracted with RNeasy Mini Kit with on-column DNase digestion (both from Qiagen). The epididymal fat tissue was homogenized with QIAzol lysis reagent, and RNA was extracted with RNeasy Lipid Tissue kit with on-column DNase digestion (all from Qiagen).

For the reverse transcription polymerase chain reaction (RT-PCR), RNA was transcribed to cDNA with Maxima First Strand cDNA Synthesis Kit (Thermo Fisher Scientific) and diluted 1:5 in RNase-free water. TaqMan Universal Master Mix and ABI Prism 7500 sequence detection system (Applied Biosystems, Foster City, CA, USA) were used for quantitative PCR. The cycling parameters were the following: incubation at 50 °C for 2 min, incubation at 95 °C for 10 min, and after that, 40 cycles of denaturation at 95 °C for 15 s and annealing and extension at 60 °C for 1 min. Glyceraldehyde 3-phosphate dehydrogenase (*Gapdh*) was used as a housekeeping gene. All other measured mRNA levels were normalized to *Gapdh* mRNA levels.

Primers and probes for *Gapdh* were the following: GCATGGCCTTCCGTGTTC (forward, 300 nM), GATGTCATCATACTTGGCAGGTTT (reverse, 300 nM), and TCGTGGATCTGACGTGCCGCC (probe, 150 nM). Primers and probes for tumor necrosis factor α (*Tnfa*) were GACCCTCACACTCAGATCATCTTCT (forward, 900 nM), CCTCCACTTGGTGGTTTGCT (reverse, 300 nM), and AAAATTCGAGTGACAAGCCTGTAGCCCA (probe, 200 nM). The sequences and concentrations were optimized according to the guideline provided by the manufacturer in TaqMan Universal PCR Master Mix Protocol part number 4304449 revision C (Applied Biosystems). The rest of the mRNA analyses were conducted using TaqMan Gene Expression Assay (Thermo Fisher Scientific, Table 2). Expression levels were calculated using the 2(−ΔΔCT) method.

### 2.5. Statistics

The results are expressed as mean ± standard error of the mean (SEM). Before statistical testing, the normality of each data group was analyzed using the D’Agostino and Pearson omnibus normality test. If the assumption of normality was not met, the data were transformed to achieve a normal distribution. One- and two-way analyses of variance (ANOVA) with Bonferroni post-test were used to analyze the statistical significance between the groups. When calculating the area under the curve (AUC) for the IPGTT, the fasting glucose level of the LF group was used as the baseline. The analyses were conducted, and the graphs were drawn with GraphPad Prism (v. 8.3.0, GraphPad Software Inc., San Diego, CA, USA).

## 3. Results

### 3.1. Weight Gain and Food Intake

**Figure 1:**
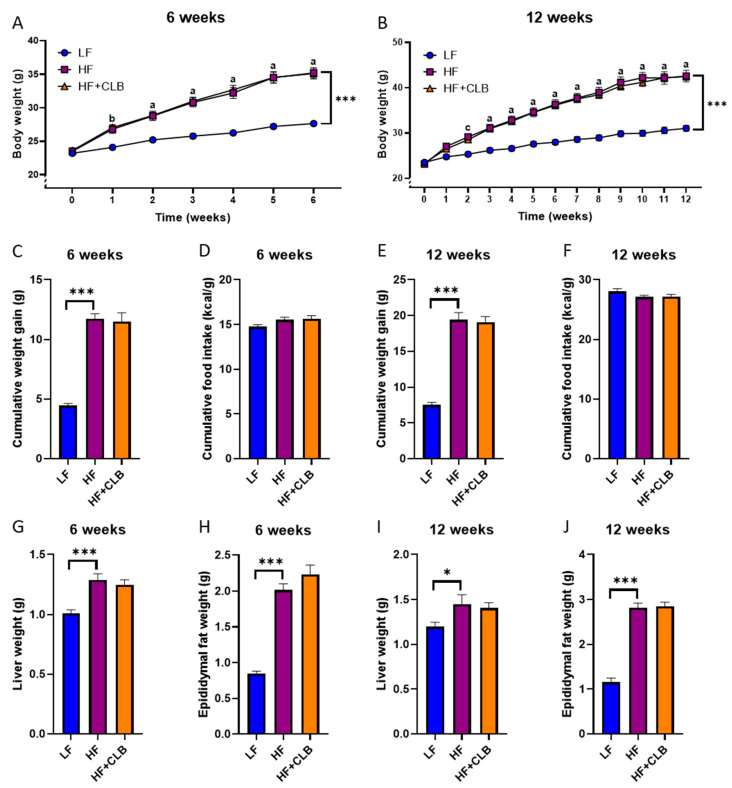
Body, liver, and epididymal fat weight gain and food intake. Mice were divided into six groups to receive either a low-fat diet (10% of energy from fat, LF), high-fat diet (46% of energy from fat, HF), or cloudberry-supplemented high-fat diet (HF+CLB) for six (**A**,**C**,**D**,**G**,**H**) or twelve (**B**,**E**,**F**,**I**,**J**) weeks. The weekly weights (**A**,**B**), the cumulative weight gain (**C**,**E**), and the weights of livers (**G**,**I**) and epididymal fat pads (**H**,**J**) are expressed as grams. The cumulative food intake (**D**,**F**) is expressed as kcal/g. The data were analyzed with two-way (mixed model) ANOVA for (**A**,**B**) and one-way ANOVA for (**C**–**J**), all with the Bonferroni post-test, * *p* < 0.05 and *** *p* < 0.001. For (**A**,**B**), the difference between LF and HF is marked with a (*p* < 0.001), b (*p* < 0.01), and c (*p* < 0.05). The values represent mean ± SEM, *n* = 12 mice per group except in D and F (*n* = 6 per group since two mice in one cage were treated as a single data point).

Seventy-two male mice were divided equally into six groups. Two groups received a low-fat diet (LF, 10% of energy from fat), two groups received a high-fat diet (HF, 46% of energy from fat), and two groups received a high-fat diet supplemented with air-dried cloudberry powder (HF+CLB). Half of the groups were treated for six weeks, and the rest were treated for twelve weeks. The weight gain trend and the cumulative weight gain in the HF groups differed from those in the LF diet groups in a statistically significant manner (Figure 1A–C,E, *p* < 0.001 for all), but there was no difference between the HF and HF+CLB groups. As obesity is often associated with hepatic inflammation and an increase in visceral fat [16], the livers and epididymal fat pads of the mice were weighed. Their weights were higher in the HF than in the LF groups, but no difference was seen between the HF and HF+CLB groups (Figure 1G–J). The food intake of the mice was recorded weekly and expressed as energy unit per body weight. There were no differences in the cumulative food intake between the groups (Figure 1D,F).

### 3.2. Liver Function and Inflammation

**Figure 2:**
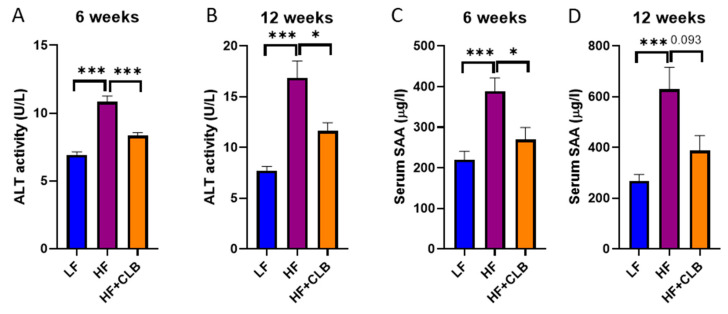
Liver function and systemic inflammation. Mice were divided into six groups to receive either a low-fat diet (10% of energy from fat, LF), high-fat diet (46% of energy from fat, HF), or cloudberry-supplemented high-fat diet (HF+CLB) for six (**A**,**C**) or twelve (**B**,**D**) weeks. The serum alanine aminotransferase (ALT) activity (**A**,**B**) is expressed as U/L. The level of serum amyloid A (SAA) in serum (**C**,**D**) is expressed as μg/L. The data were analyzed with one-way ANOVA and Bonferroni post-test, * *p* < 0.05 and *** *p* < 0.001. The values represent mean + SEM, *n* = 12 mice per group.

The average alanine aminotransferase (ALT) activity, reflecting the liver function (and when increased, inflammation/injury), at week six was 6.9 ± 0.22 U/L in the LF group, 11 ± 0.44 U/L in the HF group, and 8.3 ± 0.24 U/L in the cloudberry-supplemented HF group. The difference between the HF and LF groups and between the HF and HF+CLB groups was statistically significant (*p* < 0.001) (Figure 2A). At week twelve, the ALT values were 7.7 ± 0.39 (LF), 17 ± 1.7 (HF), and 12 ± 0.81 (HF+CLB), respectively, and the differences were statistically significant (*p* < 0.001 for HF vs. LF and *p* < 0.05 for HF vs. HF+CLB) (Figure 2B). The average serum amyloid A (SAA) level, reflecting systemic inflammation, at week six was 220 ± 21 μg/L in the LF group, 390 ± 33 μg/L in the HF group, and 270 ± 30 μg/L in the HF+CLB group. The difference between the HF and LF groups was statistically significant (*p* < 0.001), as was the difference between the HF and HF+CLB groups (*p* < 0.05) (Figure 2C). At week twelve, the SAA levels were 270 ± 24 (LF), 630 ± 87 (HF), and 390 ± 60 μg/L (HF+CLB), respectively. The HF and LF groups differed in a statistically significant manner (*p* < 0.01), but the difference between the HF and HF+CLB groups did not reach statistical significance (*p* = 0.093) (Figure 2D).

**Figure 3:**
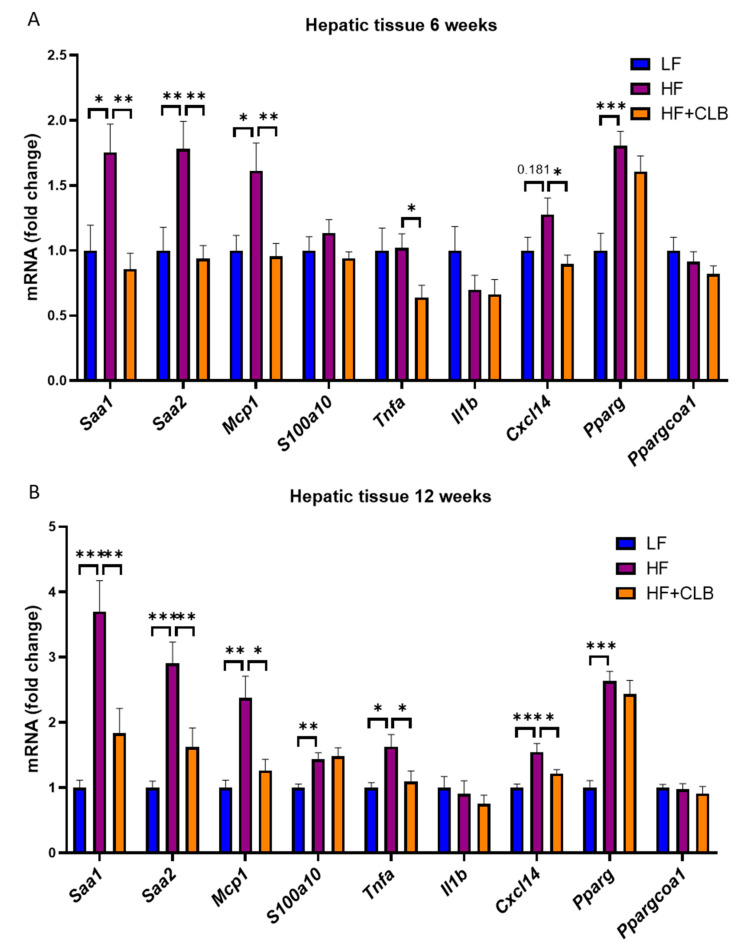
Inflammation-related factors expressed in the liver. Mice were divided into six groups to receive either a low-fat diet (10% energy from fat, LF), high-fat diet (46% of energy from fat, HF), or cloudberry-supplemented high-fat diet (HF+CLB) for six or twelve weeks. The gene expression of inflammation-related factors in the liver at weeks six (**A**) and twelve (**B**) is expressed as fold change relative to the LF group, which was set as 1. The expression levels were measured with RT-PCR and normalized to glyceraldehyde 3-phosphate dehydrogenase (*Gapdh*). The data were analyzed with one-way ANOVA and Bonferroni post-test, * *p* < 0.05, ** *p* < 0.01, and *** *p* < 0.001. The values represent mean + SEM, *n* = 12 mice per group. *Saa* = serum amyloid A; *Mcp1* = monocyte chemoattractant protein 1; *S100a* = S100 calcium-binding protein A; *Tnfa* = tumor necrosis factor α; *Il1b* = interleukin 1β; *Cxcl14* = chemokine (C-X-C) motif ligand 14; *Pparg* = peroxisome proliferator-activated receptor γ; *Ppargcoa1* = peroxisome proliferator-activated receptor γ coactivator 1α.

The expression levels of inflammation-related factors were measured in the liver (Figure 3). The expression of serum amyloid A 1 and 2 (*Saa1* and *Saa2*) and monocyte chemoattractant protein 1 (*Mcp1*) coding genes was statistically significantly higher in the HF group compared to the LF and HF+CLB groups at both timepoints. At week twelve, the expression of tumor necrosis factor α (*Tnfa*) and chemokine (C-X-C motif) ligand 14 (*Cxcl14*) was higher in the HF group compared to the LF and HF+CLB groups, and the differences were statistically significant. At week six, the difference between the HF and HF+CLB groups in *Cxcl14* and *Tnfa* expression was statistically significant (*p* < 0.05 for both), but the difference between the HF and LF groups did not reach statistical significance. However, the expression of peroxisome proliferator-activated receptor γ (*Pparg*) at both weeks and the expression of S100 calcium-binding protein 10 (*S100a10*) at week twelve differed statistically significantly between the HF and LF groups, but there was no statistically significant difference between the HF and HF+CLB groups.

**Figure 4:**
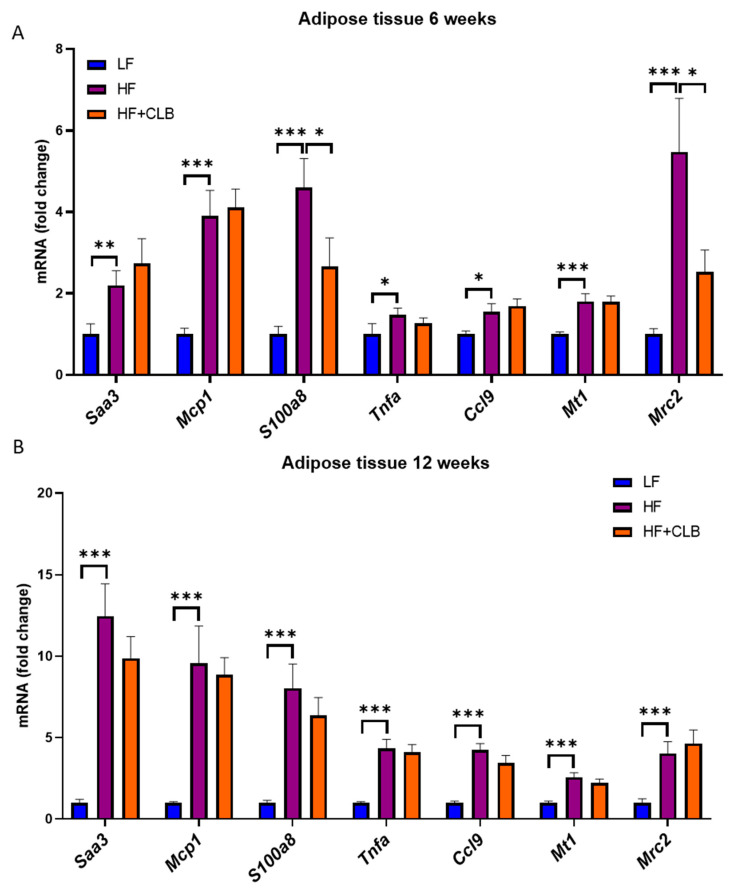
Inflammation-related factors expressed in the adipose tissue. Mice were divided into six groups to receive either a low-fat diet (10% of energy from fat, LF), high-fat diet (46% of energy from fat, HF), or cloudberry-supplemented high-fat diet (HF+CLB) for six or twelve weeks. The gene expression of inflammation-related factors in the epididymal adipose tissue at weeks six (**A**) and twelve (**B**) is expressed as fold change relative to the LF group, which was set as 1. The expression levels were measured with RT-PCR and normalized to glyceraldehyde 3-phosphate dehydrogenase (*Gapdh*). The data were analyzed with one-way ANOVA and Bonferroni post-test, * *p* < 0.05, ** *p* < 0.01, and *** *p* < 0.001. The values represent mean ± SEM, *n* = 12 mice per group. *Saa* = serum amyloid A *Mcp1* = monocyte chemoattractant protein 1; *S100a* = S100 calcium-binding protein A; *Tnfa* = tumor necrosis factor α; *Ccl9* = chemokine (C-C) motif ligand 9; *Mt1* = metallothionein 1; *Mrc2* = mannose receptor C type 2.

In addition to the liver, the adipose tissue expresses many factors related to inflammation. As the epididymal fat pads represent visceral fat that is metabolically more harmful than subcutaneous fat [5], inflammation-related factors were measured from the epididymal pads. The HF diet increased the expression of all measured factors (*Saa3*, *Mcp1*, *S100a8, Tnfa*, *Ccl9*, *Mt1*, and *Mrc2*) at weeks six and twelve in a statistically significant manner. However, the only statistically significant differences between the HF and HF+CLB groups were in the expression of *S100a8* and mannose receptor C type 2 (*Mrc2*) (*p* < 0.05 for both) at week six (Figure 4).

### 3.3. Lipids and Adipokines

**Figure 5:**
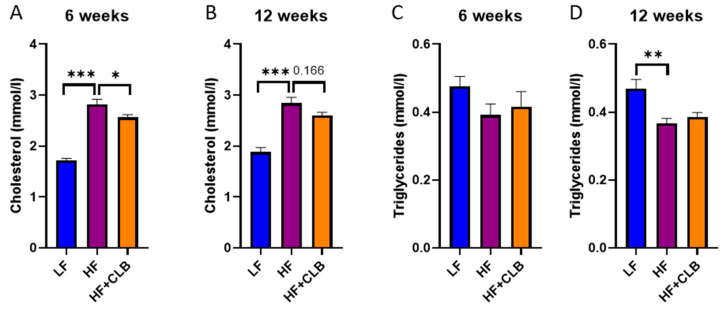
Cholesterol and triglycerides. Mice were divided into six groups to receive either a low-fat diet (10% of energy from fat, LF), high-fat diet (46% of energy from fat, HF), or cloudberry-supplemented high-fat diet (HF+CLB) for six (**A**,**C**) or twelve (**B**,**D**) weeks. The serum cholesterol levels (**A**,**B**) and the serum triglyceride levels (**C**,**D**) are expressed as mmol/L. The data were analyzed with one-way ANOVA and Bonferroni post-test, * *p* < 0.05, ** *p* < 0.01, and *** *p* < 0.001. The values represent mean + SEM, *n* = 12 mice per group.

An essential marker of lipid metabolism, cholesterol, was 1.7 ± 0.040 mmol/L in the LF group, 2.8 ± 0.10 mmol/L in the HF group, and 2.6 ± 0.058 in the HF+CLB group at week six. The difference between the HF and LF groups was statistically significant (*p* < 0.001), and so was the difference between the HF and HF+CLB groups (*p* < 0.05) (Figure 5A). At week twelve, the difference in the cholesterol levels between the HF and LF group retained statistical significance (*p* < 0.001), but only a tendency was seen between the HF and HF+CLB groups (*p* = 0.166) (Figure 5B). Another lipid metabolism marker, triglyceride, differed statistically significantly between the HF and LF groups at week twelve (*p* < 0.01) (Figure 5C,D).

Adipokines are cytokines produced mainly by the adipose tissue. Their levels in the bloodstream and expression in the epididymal fat pads and liver were measured (Table 3). The circulating adipsin and leptin levels and their expression in the adipose tissue differed between the HF and LF groups at both timepoints in a statistically significant manner. Additionally, the expression of the leptin receptor in the liver was significantly lower in the HF than in the LF group at both weeks. Cloudberry powder did not, however, cause any statistically significant difference in the measured adipokines or leptin receptor.

### 3.4. Glucose Metabolism

**Figure 6:**
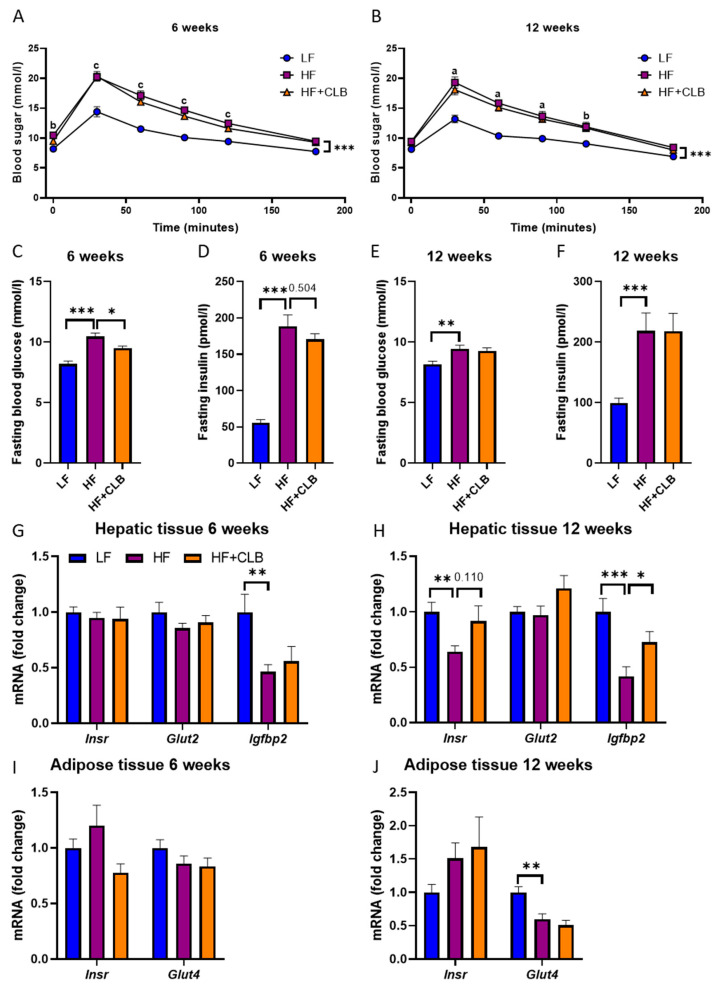
Glucose metabolism. Mice were divided into six groups to receive either a low-fat diet (10% of energy from fat, LF), high-fat diet (46% of energy from fat, HF), or cloudberry-supplemented high-fat diet (HF+CLB) for six (**A**,**C**,**D**,**G**,**I**) or twelve (**B**,**E**,**F**,**H**,**J**) weeks. A week before the end of the treatment, the fasting glucose was measured, and an intraperitoneal glucose tolerance test (IPGTT) was performed. The fasting glucose levels and the glucose levels after the glucose bolus (2 mg/g; (**A**–**C**,**E**)) are expressed as mmol/L. The fasting insulin levels (**D**,**F**) are expressed as pmol/L. The gene expression of glucose metabolism-related factors in the liver (**G**,**H**) and in the epididymal adipose tissue (**I**,**J**) is expressed as fold change relative to the LF group, which was set as 1. The expression levels were measured with RT-PCR and normalized to glyceraldehyde 3-phosphate dehydrogenase (*Gapdh*). The data were analyzed with two-way (mixed model) ANOVA for (**A**,**B**) and with one-way ANOVA for (**C**–**J**), all with the Bonferroni post-test. The area under the curve (AUC) was calculated using the average of the LF group at 0 min as baseline for testing with one-way ANOVA and Bonferroni post-test. * *p* < 0.05, ** *p* < 0.01, and *** *p* < 0.001. For (**A**,**B**), the difference between LF and HF is marked with a (*p* < 0.001), b (*p* < 0.01), and c (*p* < 0.05). The values represent mean ± SEM, *n* = 12 mice per group. *Insr* = insulin receptor; *Glut2* = glucose transporter type 2; *Glut4* = glucose transporter type 4; *Igfbp2* = insulin-like growth factor binding protein 2.

A week before the end of the treatment, i.e., on weeks five and eleven, an intraperitoneal glucose tolerance test (IPGTT) was performed to evaluate the ability of the body to metabolize excess glucose (Figure 6A,B). At week five, the average fasting glucose level in the HF group was 11 ± 0.28 mmol/L, which was higher than those in the LF (8.2 ± 0.24 mmol/L; *p* < 0.001) and HF+CLB (9.5 ± 0.19 mmol/L; *p* < 0.05) groups (Figure 6C). At week eleven, the difference in the fasting blood glucose level between the HF and LF groups remained statistically significant (*p* < 0.01), but there was no statistically significant difference between the HF and HF+CLB groups (Figure 6E).

Throughout the IPGTT (Figure 6A,B), the blood glucose levels in the HF group were higher than those in the LF group. The difference was statistically significant both at week five and at week eleven (*p* < 0.001). No statistically significant difference was seen between the HF and HF+CLB groups at either timepoint. The area under the curve (AUC) method gave similar results.

At the end of the treatment (at week six or twelve), the fasting insulin levels and the expression of genes related to glucose metabolism in the liver and in the epididymal adipose tissue were measured. Serum insulin was higher in the HF than in the LF group at both timepoints (*p* < 0.001), but cloudberry supplementation had no effect on circulating insulin levels (Figure 6D,F). At week twelve, the liver *Igfbp2* expression in the HF group was less than half (0.42 ± 0.086) of the expression in the LF group (1 ± 0.12, *p* < 0.001), and cloudberry supplementation partly prevented the decrease (0.73 ± 0.094, *p* < 0.05). At week twelve, the expression of the insulin receptor in the liver (*p* < 0.01) and the *Glut4* glucose transporter in the epididymal fat (*p* < 0.01) was also decreased by the HF diet, but those effects were not prevented by cloudberry supplementation (Figure 6G–J).

## 4. Discussion

**Figure 7:**
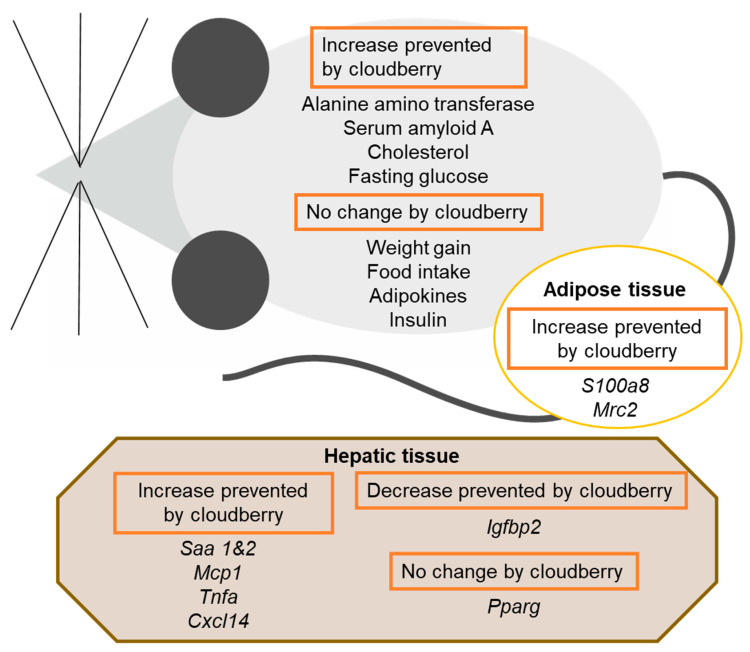
Summary of the results. Weight gain, food intake, biomarkers measured in serum, and gene expression in hepatic and adipose tissues. *Saa* = serum amyloid A; *Cxcl14* = chemokine (C-X-C) motif ligand 14; *Igfbp2* = insulin-like growth factor binding protein 2; *Mcp1* = monocyte chemoattractant protein 1; *Mrc2* = mannose receptor C type 2; *Pparg* = peroxisome proliferator-activated receptor γ; *S100a8* = S100 calcium-binding protein A; *Tnfa* = tumor necrosis factor α.

Air-dried cloudberry (*Rubus chamaemorus* L.) powder was added to a high-fat diet to study the effects of cloudberry on weight gain, liver and adipose tissue inflammation, and glucose and lipid metabolism in a mouse model of obesity. The cloudberry powder was found to prevent the rise in the liver inflammation/injury marker ALT and the acute-phase protein SAA, as well as the increased expression of inflammatory factors in the liver and adipose tissue (Figure 7). These results suggest that cloudberry can alleviate the low-grade inflammation caused by a high-fat diet and obesity. In addition, cloudberry supplementation hindered the elevation of circulating cholesterol, the rise in fasting blood glucose, and the reduction in *Igfbp2* expression in hepatic tissue.

Cloudberry belongs to the *Rosaceae* family and grows in the Northern Hemisphere, mainly in Fennoscandia, Russia, and North America [17]. It is part of the traditional diet in boreal areas such as Finland and Alaska [7,8,18]. Cloudberry and especially its seeds are a major source of ellagitannins such as ellagic acid and gallic acid [7,8]. Cloudberry also contains remarkable amounts of vitamins C and E, which are well-known for their high antioxidant capacity [19,20,21]. Ellagitannins have various biological activities; they have been reported to chelate metal ions, bind and precipitate proteins, and inhibit enzymes, and they also have antioxidant capacities [22,23]. The antioxidant effects of ellagitannins comprise direct reduction of reactive oxygen species (ROS) and lipid peroxidation, as well as an increase in antioxidative enzyme levels [24]. An example of activities other than antioxidant effects is the activation of the ERK-1/2 pathway and the stimulation of the transcription factor Nrf2 by the ellagitannin precursor 1,2,3,4,6-penta-O-galloyl-β-D-glucose. This further increases the expression of the cytoprotective and anti-inflammatory protein heme oxygenase-1 in HepG2 hepatocytes [25].

In the present study, the mice in the HF group gained more weight and performed worse in the intraperitoneal glucose tolerance test than the mice in the LF group. The weight and epididymal fat gain in the mice on the cloudberry-supplemented high-fat diet did not differ from those in the HF control group. Therefore, the differences between the HF and HF+CLB groups in other parameters, such as ALT or SAA, are not associated with the obesity or leanness of the animals, but they are most likely due to bioactive compounds in cloudberry, such as ellagitannins.

In the present study, cloudberry supplementation significantly prevented the rise in the hepatocyte inflammation/injury marker ALT. It also prevented the increase in the circulating acute-phase protein SAA induced by the high-fat diet. The expression of SAA-coding genes *Saa1* and *Saa2* in the liver was significantly lower in the HF+CLB group compared to the HF group. Although SAAs belong to the group of acute-phase proteins, their levels often also remain elevated in chronic inflammatory states [26]. The other inflammatory factors whose expression levels were lower in the HF+CLB group compared to the HF group were S100A8 and MRC-2 in the adipose tissue and CXCL-14, TNF-α, and MCP-1 in the liver. MCP-1 and TNF-α are secreted by, for instance, injured hepatocytes, injured adipocytes, and classically activated M1-type macrophages [27]. MCP-1 is the primary chemoattractant for monocytes and macrophages in inflamed hepatic tissue [27,28]. In a study of *Mcp1* knock-out mice, a high-fat diet was unable to induce hepatic steatosis and insulin resistance, and adipose tissue produced less TNF-α [29]. TNF-α accelerates the inflammatory reaction by activating MAP kinase and NF-κB-mediated signaling pathways [28]. Suppressing NF-κB signaling is among the actions of vitamins C and E [20,30], and it has also been proposed as the main mechanism of the anti-inflammatory effects of ellagitannins [22,31]. In addition, the ellagitannin derivative punicalagin favors macrophage polarization towards the anti-inflammatory M2 phenotype [32]. CXCL-14 is a chemotactic factor that attracts immune cells, mainly monocytes and dendritic cells. CXCL-14 supports the immune system [33], but in the metabolic context, the role of CXCL-14 in obesity is complicated [34,35].

In the present study, the expression levels of *S100a8* and *Mrc2* genes in the adipose tissue were increased in mice on the high-fat diet, and the effect was prevented by cloudberry supplementation. *S100a8* is expressed in neutrophils and monocytes. Together with S100A9, S100A8 protein forms a heterodimer that recruits macrophages to adipose tissue, promoting inflammation [36]. MRC-2 belongs to the mannose receptor family, and the *Mrc2* gene is expressed in macrophages and endothelial cells [37]. The main function of MRC-2 is linked to the assistance of macrophages to endocytose collagens [38]. In the present results, however, the reduction in *Mrc2* expression in the adipose tissue is likely to reflect the number of macrophages in the tissue. Accordingly, two macrophage-related cytokines were less abundant in the adipose tissue in the HF+CLB group compared to the HF group. Additionally, cloudberry supplementation significantly prevented the rise in circulating cholesterol but did not affect any of the adipokines measured. As all of the cloudberry effects in the adipose tissue were observed at week six but no longer at week twelve, the result indicates that cloudberry may retard but not fully prevent the development of adipose tissue dysfunction.

Overweight and excess energy intake are risk factors for the development of insulin resistance. Ellagitannins inhibit the activity of the enzymes α-glucosidase, pancreatic lipase, and salivary α-amylase and the secretion of resistin; they also stimulate insulin secretion and glucose transport [22,23,24]. These are all possible antihyperglycemic mechanisms. At the cellular level, cloudberry extract has also been shown to prevent glucose uptake by HepG2 hepatocytes [39]. However, when ellagitannins have been studied in animal models of hyperglycemia and in hyperglycemic humans, the results have been less clear [24]. In the present study, the mice in the HF group had significantly higher fasting glucose levels and handled intraperitoneally administered glucose worse in the IPGTT than the mice in the LF group. Cloudberry supplementation lowered the level of fasting glucose and partly prevented the reduction in the expression of *Igfbp2* in the liver. Insulin-like growth factor binding protein-2 (IGFBP-2) correlates negatively with body weight and insulin resistance. In hepatic tissue, insulin downregulates the expression of *Igfbp2* [40].

Biochemically, ellagitannins are a vast group of hydrolyzable tannins [23]. The environment where the cloudberry plant grows affects the polyphenolic content of its berries, resulting in some variation in the ellagitannin composition between different batches of berries or berry products [41]. Despite that, single ellagitannins and ellagitannin-containing foods or their derivatives have been shown to have anti-inflammatory and anti-diabetic effects in murine models [24,31]. Polyphenols and antioxidants affect each other’s bioavailability, and they may function synergistically [42]. Cloudberry contains significant amounts of vitamins C and E, which are known to enhance the antioxidant properties of other antioxidants [20,21]. Thus, it is likely that vitamins C and E and ellagitannins act synergistically when administered at the same time. Due to the synergy, studying combinations of different polyphenols or polyphenol-containing plant preparations over single compounds is advisable. As polyphenols and their combinations vary from one berry species to another and the majority of ellagitannin studies in the literature have been conducted on pomegranate and raspberry products [22,24,31], there is a need for cloudberry studies.

To our knowledge, there are only two previous studies on cloudberry in a dietary mouse model and few clinical studies where cloudberry has been among the berries studied. In the animal studies, cloudberry extract had no effect on weight gain but hindered the development of hyperinsulinemia [43,44]. The clinical studies concentrated on glucose and lipid metabolism with promising results [45,46]. The present study is the first one using air-dried cloudberry and the only one where the effects of cloudberry on liver and adipose tissue inflammation were studied. Therefore, it extends the previous knowledge on the health-promoting effects of cloudberry on adverse inflammatory and metabolic changes in high-fat-diet-induced obesity.

Despite the current promising results, further studies are needed to confirm whether the findings can be translated to humans. The use of a rodent model enables wider sampling and better control of potentially confounding nutritional and environmental factors than human studies. On the other hand, metabolic and other differences between the two species may affect the applicability of the results in the clinical context. The choice of the parameters studied in the present study was based on the literature discussed above and our previous experience with the high-fat diet model [47,48]. However, it is likely that we were unable to cover all of the essential tissues and parameters.

## 5. Conclusions

In the present study, the effects of cloudberry supplementation were studied in a high-fat-diet-induced obesity model in mice. Cloudberry supplementation had no effect on weight gain, but it prevented the rise in circulating ALT and SAA, as well as the expression of multiple inflammatory factors in hepatic and adipose tissues. Cloudberry supplementation also decelerated the development of hypercholesterolemia and hyperglycemia. These results encourage further studies on the effects of cloudberry to support the use of cloudberries as a part of a healthy diet.

## Figures and Tables

**Table 1:** The nutritional composition of the experimental diets.

Nutrient	Low-Fat Diet (LF)	High-Fat Diet (HF)	Cloudberry Supplemented High-Fat Diet (HF+CLB)
**Calculated energy from protein, carbohydrate, and fat per gram of diet (kcal/g)**			
Protein	0.7	0.8	0.8
Carbohydrate	2.7	1.6	1.6
Fat	0.4	2.0	2.1
Total Energy	3.7	4.6	4.4
**Calculated Energy (kcal %)**			
Protein	29	18	18
Carbohydrate	72	36	36
Fat	10	46	46
Total Energy	100	100	100
**Ingredients (g)**			
Cloudberry powder (CLB, g)	0	0	180 ^1^
Casein (protein)	200	200	180 (+20 from CLB), total 200
L-Cysteine	3	3	3
Corn Starch	448	68	63 (+4 from CLB), total 67
Maltodextrin 10	75	100	100
Glucose	28	28	6 (+22 from CLB), total 28
Fructose	38	38	11 (+27 from CLB), total 38
Sucrose	107	107	107
Cellulose (insoluble fiber)	54	54	0 (+54 from CLB), total 54
Inulin (soluble fiber)	12	12	0 (+12 from CLB), total 12
Soybean oil	25	25	8 (+11 from CLB), total 19
Lard	20	178	182
Mineral Mix S10026	10	10	10
DiCalcium Phosphate	13	13	13
Calcium Carbonate	6	6	6
Potassium Citrate	17	17	17
Vitamin Mix V10001	10	10	10
Choline Bitartrate	2	2	2

^1^ Nutrient content of 100 g cloudberry powder: fat 7 g, carbohydrates 33.5 g (of which sugars 28 g), fiber 37 g, and protein 9.7 g.

**Table 2:** TaqMan Gene Expression Assays.

Gene	Abbreviation	Assay ID
Adiponectin	*Adipoq*	Mm00456425_m1
Adipsin	*And*	Mm01143935_g1
Chemokine (C-C motif) ligand 9	*Ccl9*	Mm00441260_m1
Chemokine (C-X-C) motif ligand 14	*Cxcl14*	Mm00444699_m1
Glucose transporter type 2	*Glut2*	Mm00446229_m1
Glucose transporter type 4	*Glut4*	Mm00436615_m1
Insulin-like growth factor binding protein 2	*Igfbp2*	Mm00492632_m1
Insulin receptor	*Insr*	Mm01211875_m1
Interleukin 1β	*Il1b*	Mm00434228_m1
Leptin	*Lep*	Mm00434759_m1
Leptin receptor	*Lepr*	Mm00440181_m1
Mannose receptor C type 2	*Mrc2*	Mm00485184_m1
Metallothionein 1	*Mt1*	Mm00496660_g1
Monocyte chemoattractant protein 1	*Mcp1*	Mm00441242_m1
Peroxisome proliferator-activated receptor γ	*Pparg*	Mm01184322_m1
Peroxisome proliferator-activated receptor γ coactivator 1 α	*Ppargc1a*	Mm01208835_m1
Resistin	*Retn*	Mm00445641_m1
S100 calcium-binding protein A8	*S100a8*	Mm00496696_g1
S100 calcium-binding protein A10	*S100a10*	Mm00501458_g1
Serum amyloid A1	*Saa1*	Mm00656927_g1
Serum amyloid A2	*Saa2*	Mm04208126_mH
Serum amyloid A3	*Saa3*	Mm00441203_m1

**Table 3:** Serum adipokine levels and adipokine expression in the epididymal fat pads and in the liver. Mice were divided into six groups to receive either a low-fat diet (10% of energy from fat, LF), high-fat diet (46% of energy from fat, HF), or cloudberry-supplemented high-fat diet (HF+CLB) for six or twelve weeks. The adipokine levels in the serum were measured by enzyme-linked immunosorbent assay (ELISA). The expression of adipokines and leptin receptor in the epididymal adipose tissue and in the liver was measured with RT-PCR and normalized to glyceraldehyde 3-phosphate dehydrogenase (*Gapdh*); mean expression levels in the LF group were set as 1, and the other values are given relative to that value. The data were analyzed with one-way ANOVA and Bonferroni post-test. The values represent mean ± SEM, *n* = 12 mice per group; ns = not significant (*p* > 0.05). Blue color indicates a decrease in HF vs. LF group, and green color indicates an increase in HF vs. LF group.

Adipokine	Week	LF	HF	HF+CLB	HF vs. LF *p*-Value	HF vs. HF+CLB *p*-Value
Serum						
Adiponectin (mg/L)	6	7.4 ± 0.20	6.2 ± 0.12	6.1 ± 0.10	<0.001	ns
	12	6.6 ± 0.12	6.4 ± 0.20	6.1 ± 0.11	ns	ns
Adipsin (mg/L)	6	12 ± 0.38	7.5 ±0.33	7.3 ±0.43	<0.001	ns
	12	10 ± 0.38	6.9 ± 0.55	6.2 ± 0.27	<0.001	ns
Leptin (μg/L)	6	3.5 ± 0.29	26 ± 2.3	24 ± 2.3	<0.001	ns
	12	6.9 ± 1.1	39 ± 4.5	38 ± 3.3	<0.001	ns
Resistin (μg/L)	6	18 ± 0.69	17 ± 0.58	17 ± 0.39	ns	ns
	12	19 ± 1.1	18 ± 0.63	17 ± 1.1	ns	ns
Adipose tissue, gene expression						
Adiponectin	6	1 ± 0.037	1.0 ± 0.093	1.0 ± 0.060	ns	ns
	12	1 ± 0.049	0.85 ± 0.12	0.72 ± 0.094	ns	ns
Adipsin	6	1 ± 0.071	0.57 ± 0.048	0.59 ± 0.040	<0.001	ns
	12	1 ± 0.077	0.41 ± 0.081	0.41 ± 0.061	<0.001	ns
Leptin	6	1 ± 0.094	3.8 ± 0.35	3.7 ± 0.24	<0.001	ns
	12	1 ± 0.15	2.5 ± 0.22	2.4 ± 0.21	<0.001	ns
Leptin receptor	6	1 ± 0.10	0.80 ± 0.11	0.67 ± 0.097	ns	ns
	12	1 ± 0.18	0.87 ± 0.19	0.95 ± 0.37	ns	ns
Resistin	6	1 ± 0.053	0.89 ± 0.081	0.99 ± 0.10	ns	ns
	12	1 ± 0.063	0.54 ± 0.047	0.54 ± 0.084	<0.001	ns
Hepatic tissue, gene expression						
Adipsin	6	1 ± 0.38	9.3 ± 1.6	7.6 ± 1.4	<0.001	ns
	12	1 ± 0.31	30 ± 6.9	53 ± 15	<0.001	ns
Leptin receptor	6	1 ± 0.12	0.18 ± 0.0091	0.092 ± 0.012	<0.001	ns
	12	1 ± 0.13	0.19 ± 0.027	0.12 ± 0.012	<0.001	ns

## Data Availability

All of the data are presented in the manuscript.
